# Surgical Experience Disparity Between Male and Female Surgeons in Japan

**DOI:** 10.1001/jamasurg.2022.2938

**Published:** 2022-07-27

**Authors:** Emiko Kono, Urara Isozumi, Sachiyo Nomura, Kae Okoshi, Hiroyuki Yamamoto, Hiroaki Miyata, Itaru Yasufuku, Hiromichi Maeda, Junichi Sakamoto, Kazuhisa Uchiyama, Yoshihiro Kakeji, Kazuhiro Yoshida, Yuko Kitagawa

**Affiliations:** 1 Department of General and Gastroenterological Surgery, Osaka Medical and Pharmaceutical University, Takatsuki, Japan; 2 Department of Healthcare Quality Assessment, Graduate School of Medicine, The University of Tokyo, Tokyo, Japan; 3 Department of Gastrointestinal Surgery, Graduate School of Medicine, The University of Tokyo, Tokyo, Japan; 4 Department of Surgery, Japan Baptist Hospital, Kyoto, Japan; 5 Department of Gastroenterological and Pediatric Surgery, Gifu University School of Medicine, Gifu, Japan; 6 Department of Surgery, Kochi Medical School, Nankoku, Japan; 7 Tokai Central Hospital, Kakamigahara, Japan; 8 Database Committee, The Japanese Society of Gastroenterological Surgery, Tokyo, Japan; 9 The Japanese Society of Gastroenterological Surgery, Tokyo, Japan

## Abstract

**Importance:**

Women are vastly underrepresented in surgical leadership and management in Japan. The lack of equal opportunities for surgical training is speculated to be the main reason for this disparity; however, this hypothesis has not been investigated thus far.

**Objective:**

To examine gender disparity in the number of surgical experiences among Japanese surgeons.

**Design, Setting, and Participants:**

This retrospective, multicenter cross-sectional study used data from the National Clinical Database, which contains more than 95% of all surgical procedures in Japan. Participants included male and female gastroenterological surgeons who performed appendectomy, cholecystectomy, right hemicolectomy, distal gastrectomy, low anterior resection, and pancreaticoduodenectomy between January 1, 2013, and December 31, 2017.

**Exposures:**

Differences in the number of surgical experiences between male and female surgeons.

**Main Outcomes and Measures:**

The primary outcomes were the total number of operations and number of operations per surgeon by gender and years of experience. Data were analyzed from March 18 to August 31, 2021.

**Results:**

Of 1 147 068 total operations, 83 354 (7.27%) were performed by female surgeons and 1 063 714 (92.73%) by male surgeons. Among the 6 operative procedures, the percentage of operations performed by female surgeons were the highest for appendectomy (n = 20 648 [9.83%]) and cholecystectomy (n = 41 271 [7.89%]) and lowest for low anterior resection (n = 4507 [4.57%]) and pancreaticoduodenectomy (n = 1329 [2.64%]). Regarding the number of operations per surgeon, female surgeons had fewer surgical experiences for all 6 types of operations in all years after registration, except for appendectomy and cholecystectomy in the first 2 years after medical registration. The largest gender disparity for each surgical procedure was 3.17 times more procedures for male vs female surgeons for appendectomy (at 15 years after medical registration), 4.93 times for cholecystectomy (at 30-39 years), 3.65 times for right hemicolectomy (at 30-39 years), 3.02 times for distal gastrectomy (at 27-29 years), 6.75 times for low anterior resection (at 27-29 years), and 22.2 times for pancreaticoduodenectomy (at 30-39 years).

**Conclusions and Relevance:**

This cross-sectional study found that female surgeons had less surgical experience than male surgeons in Japan, and this gap tended to widen with an increase in years of experience, especially for medium- and high-difficulty operations. Gender disparity in surgical experience needs to be eliminated, so that female surgeons can advance to leadership positions.

## Key Points

**Question:**

Does gender disparity exist in the number of surgical experiences among male and female surgeons in Japan?

**Findings:**

In this cross-sectional study covering 1 147 068 total operations in 6 surgical fields performed between 2013 and 2017, surgical experience was classified by surgeons’ gender and years of experience. The number of operations per surgeon was lower for female compared with male surgeons, except in the first 2 years after medical registration; this gender gap widened as the difficulty level of surgery increased.

**Meaning:**

The findings indicate a marked disparity in the surgical experience of female and male surgeons in Japan.

## Teaser

This cross-sectional study used data from the National Clinical Database in Japan to examine gender disparity in the number of surgical experiences among male and female surgeons.

## Introduction

The “glass ceiling” refers to the invisible barrier that prevents women from advancing to managerial and executive positions in organizations, even when they succeed in fields that had traditionally been dominated by men.^1^ Globally, although the percentage of female surgeons has been increasing, gender disparity still exists in the field, with fewer women in leadership roles.^2,3,4,5^

According to the Statistics of Physicians, Dentists, and Pharmacists by the Ministry of Health, Labor, and Welfare, Japan had a total of 32 448 surgeons in 2006; however, this number dropped sharply to 13 751 in 2018, resulting in a serious shortage of surgeons in the country. Furthermore, the number of female surgeons decreased from 1381 in 2006 to 853 in 2018, although they occupied a greater proportion of the workforce in 2018 (6.2% vs 4.2% in 2006). Moreover, in both years, the largest number of female surgeons were aged 30 to 34 years; this number gradually decreased as the age group increased, with very few women in leadership positions.^6,7,8,9^ Surgical training programs in Japan require graduates to complete a 2-year structured postgraduate general clinical training program. Thereafter, prospective surgeons are trained in a 3-year surgical residency and then take the Board of Surgery certification examination. During the 3-year surgical residency, the residents choose subspecialties, such as general and digestive surgery, cardiovascular surgery, pediatric surgery, and breast surgery.^10^ Although this choice is essentially made by the residents, supervisors have a strong influence, and the resident’s choice is sometimes not accepted. After passing the certification examination, surgeons work under the surgery director who allocates the surgeries.

In Japanese culture, a strong belief that women should play a central role in housework and childcare makes it difficult for women to build successful careers.^11^ Both female and male surgeons are expected to acquire a similar, certain level of surgical skill and play a leading role in surgical practice. However, to our knowledge, there has been no detailed study of surgical training for female surgeons in Japan. In other countries, there have been some reports on the disparity in surgical training between male and female surgeons, but it is still unclear whether they are sufficiently trained as surgeons for all years of experience. Because surgical experience has a significant impact on a surgeon’s career, identifying differences in surgical experience between male and female surgeons has important implications for examining the lack of female surgeons in leadership positions. The purpose of this study was to examine gender disparity in the surgical experience of surgeons in Japan, using the National Clinical Database (NCD) containing more than 95% of all operations performed in Japan,^12,13^ and to consider the implications of and countermeasures against this disparity.

## Methods

This multicenter cross-sectional study was approved by the ethics committees of Gifu University and Osaka Medical and Pharmaceutical University. An email was sent to JSGS members once a month from November 2019 to May 2020 regarding the use of member information for NCD research and offering the opportunity to refuse participation. This study followed the Strengthening the Reporting of Observational Studies in Epidemiology (STROBE) reporting guideline.

Among the operations performed by the members of The Japanese Society of Gastroenterological Surgery (JSGS) between January 1, 2013, and December 31, 2017, the following elective surgeries were selected for this cross-sectional study: appendectomy and cholecystectomy, defined as low-difficulty surgeries by the JSGS training curriculum for gastroenterological surgeons; right hemicolectomy and distal gastrectomy, defined as medium-difficulty surgeries; and low anterior resection and pancreaticoduodenectomy, defined as high-difficulty surgeries.

Data on the total number of operations performed, surgeon’s medical registration number, date of registration, and expected surgical mortality (defined by the NCD as in-hospital deaths within 90 days after surgery or any death up to 30 days after surgery) were collected from the NCD. Cholecystectomy and appendectomy were excluded from the surgical outcome analysis because there were no data on the surgical mortality for these procedures. Surgeons’ years of experience was calculated as the number of years from the date of medical registration. Gender information was obtained by matching the medical registration number with the JSGS member records that contain gender information. The number of years from the date of registration was divided into 1-year increments up to 20 years after medical registration, 3-year increments for 20 to 29 years, 10-year increments for 30 to 39 years, and no increment after 40 years.

### Statistical Analysis

The primary outcome was the number of operations per surgeon, categorized by gender and years after registration. The number of operations performed per female surgeon was calculated as follows:

*X* = *X* (2013) + *X* (2014) + *X* (2015) + *X* (2016) + *X* (2017),

where *X* (year) is the number of surgeries performed by female surgeons in their *Z*^th^ year after registration in that year divided by the number of female surgeons in their *Z*^th^ year after registration in that year. The number of surgeries per male surgeon was calculated in the same way.

The secondary outcome was the number and percentage of operations performed by male and female surgeons and the proportion of high-risk surgeries performed by the surgeons, categorized by gender and years after registration. High-risk surgeries, as defined by the NCD risk estimation system, included those in the top 25% for predicted surgical mortality within 30 days. A risk calculator, built within the NCD, was used to calculate the predicted risk by entering the necessary preoperative information into the module on the website.^14,15,16,17^ Stata, version 16 (StataCorp LLC) and Excel (Office Professional Plus 2019; Microsoft) were used for data handling, analyses, and visualizations. Data analysis was conducted from March 18 to August 31, 2021.

## Results

### Number and Percentage of Female Surgeons in the JSGS by Years of Experience

**Figure 1.**
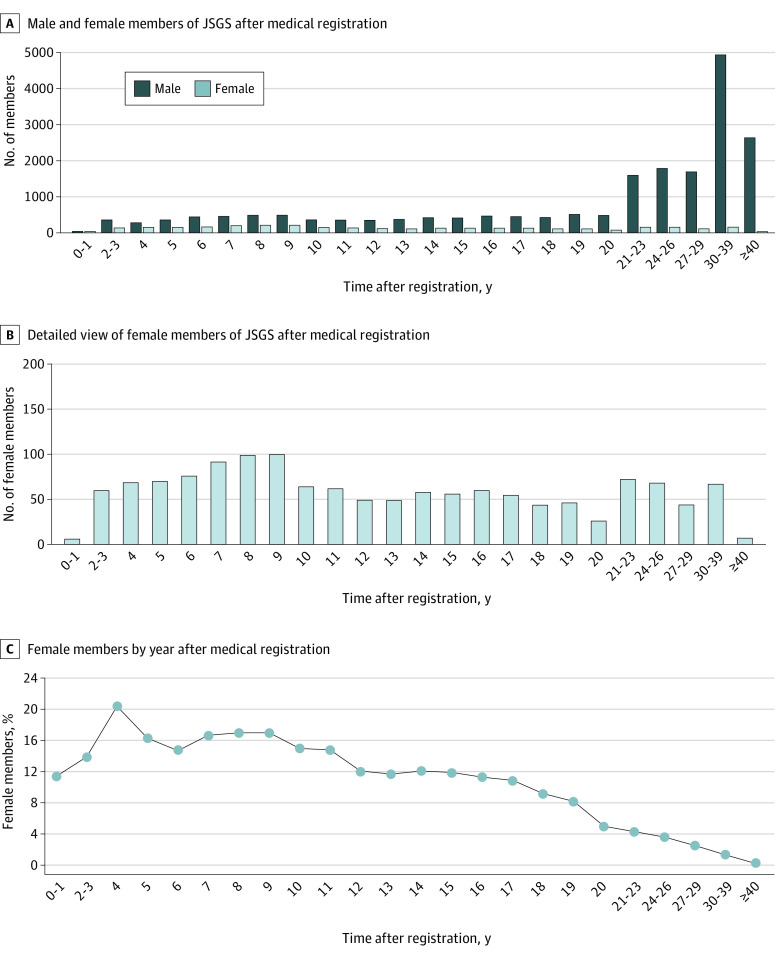
The Japanese Society of Gastrointestinal Surgery Membership

The total number of JSGS members in 2017 was 21 425, with 1375 (6.4%) female and 20 050 (93.6%) male surgeons. Classified by years since registration of medical license, there was no difference in the number of male surgeons in the different year groups, whereas the number of female surgeons tended to be higher in the first few years just after the date of registration (Figure 1A and B). The highest percentage of female surgeons (20.4%) was found in the fourth-year group (Figure 1C).

### Number and Percentage of Operations by Male and Female Surgeons

Of 1 147 068 total operations, 83 354 (7.27%) were performed by female surgeons and 1 063 714 (92.73%) by male surgeons. The most frequent operation performed by both male and female gastroenterological surgeons was cholecystectomy (523 195 total operations), followed by appendectomy (210 089 operations) and distal gastrectomy (166 235 operations) (Table 1). The proportion of operations performed by female surgeons was 9.83% (n = 20 648) for appendectomy, 7.89% (n = 41 271) for cholecystectomy, 6.51% (n = 6417) for right hemicolectomy, 5.52% (n = 9182) for distal gastrectomy, 4.57% (n = 4507) for low anterior resection, and 2.64% (n = 1329) for pancreaticoduodenectomy. Thus, female surgeons performed a larger proportion of the low-difficulty surgeries and smaller proportion of the high-difficulty surgeries.

**Table 1.** Operations Performed by Male and Female Surgeons in Japan

Procedure	Total No.	No. (%)	
		Female	Male
Appendectomy	210 089	20 648 (9.83)	189 441 (90.17)
Cholecystectomy	523 195	41 271 (7.89)	481 924 (92.11)
Right hemicolectomy	98 525	6417 (6.51)	92 108 (93.49)
Distal gastrectomy	166 235	9182 (5.52)	157.053 (94.48)
Low anterior resection	98 668	4507 (4.57)	94 161 (95.43)
Pancreaticoduodenectomy	50 356	1329 (2.64)	49 027 (97.36)
No. of surgeons registered in the JSGS (2017)	21 425	1375 (6.42)	20 050 (93.58)

Abbreviation: JSGS, The Japanese Society of Gastroenterological Surgery.

### Number of Operations According to Gender and Years of Experience

#### Low-Difficulty Surgery: Appendectomy and Cholecystectomy

**Figure 2.**
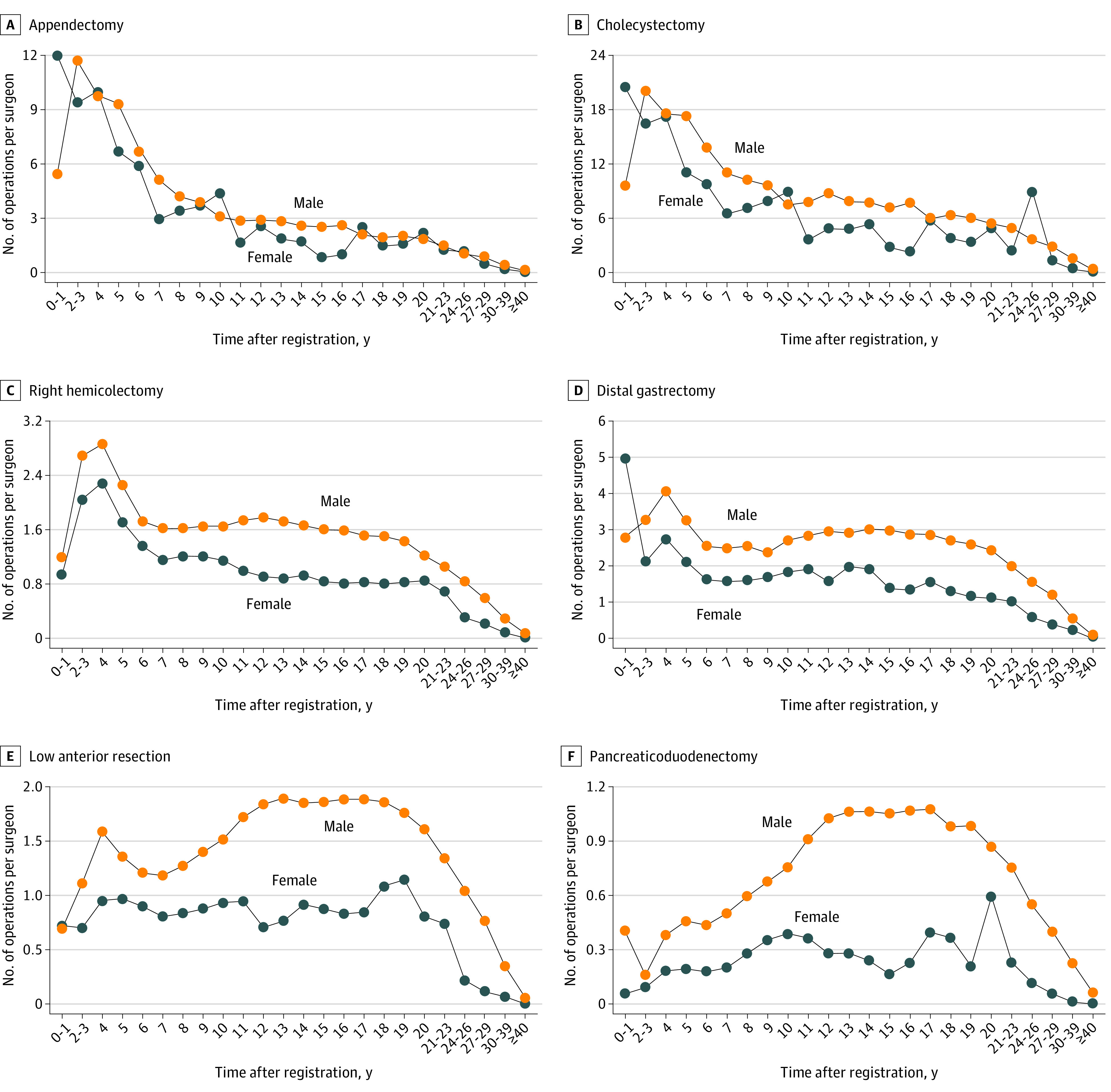
Number of Operations per Surgeon by Gender and Groups of Years After Medical Registration

The number of operations performed were classified by the surgeons’ gender and years of experience, as shown in Table 2; the number of operations per surgeon for each gender and year group is shown in Figure 2. During the first 2 years after registration, female surgeons performed more low-difficulty operations than male surgeons (appendectomy: 2.25 times more; cholecystectomy: 2.17 times more). Thereafter, male surgeons performed more operations for all procedures than female surgeons in almost all groups of years after registration. The difference between male and female surgeons for low-difficulty operations was smaller than that for medium- and high-difficulty surgeries. The difference between male and female surgeons was less than 1.5 times for approximately 60% of the total operations (less than 2 times for about 80%). The gender gap based on years of experience was also small. The largest disparities were found at 15 years after registration for appendectomy (3.17 times more operations performed by male vs female surgeons) and at 30 to 39 years after registration for cholecystectomy (4.93 times more operations performed by male vs female surgeons).

**Table 2.** Number of Operations Categorized by Surgeons’ Gender and Years After Medical Registration

	No. of operations																							
	0-1 y	2-3 y	4 y	5 y	6 y	7 y	8 y	9 y	10 y	11 y	12 y	13 y	14 y	15 y	16 y	17 y	18 y	19 y	20 y	21-23 y	24-26 y	27-29 y	30-39 y	≥40 y
Appendectomy																								
Female	216	3314	3564	2578	2286	1087	1138	1111	1234	423	658	460	400	182	191	439	208	190	222	365	268	82	32	0
Male	874	21 329	16 658	18 596	14 245	10 265	8112	7251	5592	5328	5637	5785	5305	5374	5710	4635	4453	4893	4690	11 706	8409	6804	7190	600
Cholecystectomy																								
Female	367	5798	6132	4263	3790	2402	2368	2405	2496	941	1237	1193	1281	631	437	1010	537	403	503	685	2123	210	59	0
Male	1540	36 522	29 763	34 477	28 474	21 915	19 784	17 994	13 636	14 536	17 094	15 967	15 987	15 144	16 977	13 346	14 753	14 752	13 782	39 855	29 838	23 182	30 538	2068
Right hemicolectomy																								
Female	17	736	827	668	532	436	412	374	327	263	237	225	227	194	164	147	117	104	88	199	72	36	15	0
Male	197	4957	4925	4530	3594	3272	3178	3132	3069	3305	3529	3577	3475	3482	3558	3452	3524	3558	3142	8579	6956	4788	5752	557
Distal gastrectomy																								
Female	89	748	982	823	630	588	539	518	519	504	405	501	465	319	269	279	188	143	114	304	139	70	46	0
Male	459	5930	6910	6419	5256	5003	4948	4430	4979	5340	5801	5966	6251	6445	6388	6480	6272	6430	6252	16 201	12 948	9866	11 232	847
Low anterior resection																								
Female	13	250	341	376	350	302	282	270	265	250	183	193	223	203	167	150	158	143	83	220	52	20	13	0
Male	113	2039	2709	2716	2501	2382	2482	2632	2799	3247	3624	3908	3851	4027	4190	4280	4347	4360	4146	10 930	8844	6312	7176	546
Pancreaticoduodenectomy																								
Female	1	32	65	75	69	75	94	109	110	96	72	71	58	38	46	70	53	26	61	68	28	10	2	0
Male	66	291	648	916	902	1008	1155	1268	1390	1720	2017	2183	2206	2270	2371	2434	2279	2433	2236	6153	4559	3314	4656	552

#### Medium-Difficulty Surgery: Right Hemicolectomy and Distal Gastrectomy

Female surgeons performed more distal gastrectomies than did male surgeons for the first 2 years after registration (1.77 times more). Male surgeons performed more right hemicolectomies than female surgeons in all groups of years after registration. The differences between male and female surgeons for medium-difficulty operations were in the middle of the differences for low- and high-difficulty surgeries and increased with years of experience. The number of male surgeons performing right hemicolectomy was 1.2 to 1.4 times that of female surgeons for the first 10 years but increased to 1.7 to 2.0 times thereafter and was more than 2.7 times at 24 years after registration. Male surgeons performed distal gastrectomy 1.4 to 1.6 times more than female surgeons 14 years after registration; however, this number increased to approximately 2.0 times thereafter and more than doubled 24 years after registration.

The largest gender disparities were found at 30 to 39 years after registration, when male surgeons performed a right hemicolectomy 3.65 times more often than female surgeons; and at 27 to 29 years after registration, when male surgeons performed a distal gastrectomy 3.02 times more often than female surgeons.

#### High-Difficulty Surgery: Low Anterior Resection and Pancreaticoduodenectomy

For both low anterior resection and pancreaticoduodenectomy, male surgeons performed more procedures than female surgeons in all groups of years after registration. The difference between male and female surgeons for high-difficulty surgeries was larger than for low- and medium-difficulty surgeries and increased with years of experience. The number of male surgeons performing low anterior resection was 1.3 to 1.8 times that of female surgeons for the first 11 years after registration and more than 2 times thereafter. From 18 to 23 years after registration, male surgeons performed less than 2 times more than female surgeons, but after 24 years, they performed approximately 5 times the number of anterior resections. The number of pancreaticoduodenectomies performed by male surgeons was about 7 times that of female surgeons in the first 2 years after registration; thereafter, it was approximately 2 times for up to 10 years, 2.5 to 7.0 times for 11 to 29 years, and 22.2 times for 30 to 39 years after registration. The largest gender disparity was found at 27 to 29 years, with male surgeons performing low anterior resection 6.75 times more than female surgeons; and at 30 to 39 years, with male surgeons performing pancreaticoduodenectomy 22.2 times more than female surgeons.

### Proportion of High-risk Surgery

**Figure 3.**
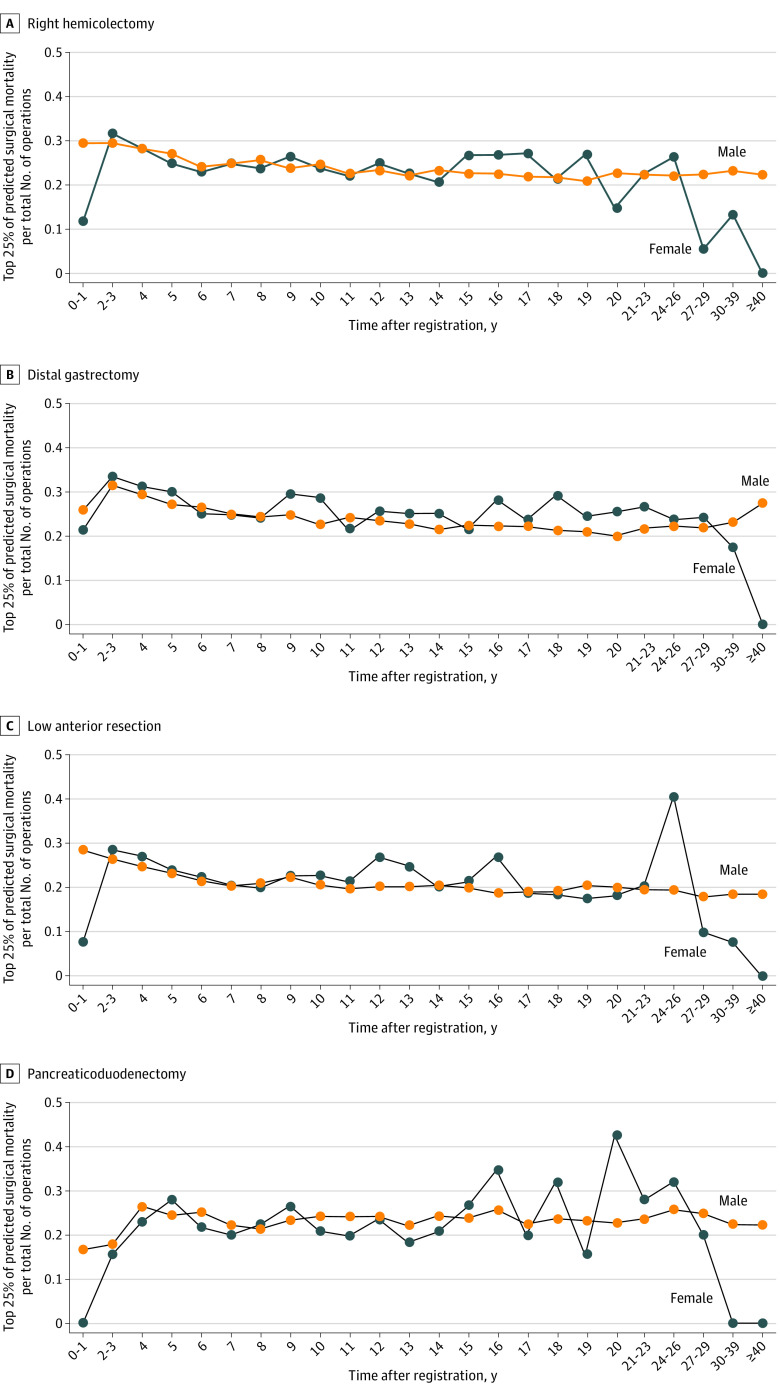
Proportion of Operations in the Top 25% for Predicted Surgical Mortality by Total Number of Operations Performed by Surgeons Grouped by Gender and Years After Medical Registration

The proportion of high-risk surgery performed by male and female surgeons, classified by years after registration, is shown in Figure 3. High-risk surgeries ranged between a proportion of 0.2 and 0.3 of the total surgeries performed by the surgeons in all groups of years after registration, except the first 2 years. There were no gender differences in the rate of high-risk surgeries; however, the numbers varied more among female surgeons, especially in the later years after registration, possibly owing to the small number of female surgeons in these year groups.

## Discussion

To our knowledge, this is the first study to classify the number of gastroenterological surgeries performed in Japan by the surgeons’ gender and years of experience. The findings revealed a marked disparity between female and male surgeons in terms of their surgical experiences. These results are important for identifying and alleviating discrimination against female surgeons during surgical training in Japan.

In Japan, junior residents do not choose a department for 2 years after registration and perform rotations through several designated departments.^18^ The large number of female junior residents observed in low- and medium-difficulty operations may be due to the recent increase in female surgeons among new members of the JSGS. It is possible that the attending surgeons actively recruited female junior residents for gastroenterological surgery by providing operative opportunities for them. On the other hand, in pancreaticoduodenectomy, male junior residents performed the surgery 7 times more than female junior residents, which may be due to gender bias of the attending surgeons who allocate surgical assignments. It is also possible that female junior residents are reluctant to undergo surgical training for high-difficulty procedures for a variety of reasons, including long working hours, difficulty in balancing work and family, and absence of role models.^19,20^

After junior residency training, senior residents receive training in the specialty of their choice. In the Department of Surgery, senior residents rotate through several surgical specialties for 3 years, depending on their program.^21^ In the current study, it is noteworthy that male residents had more surgical experience than female residents for all 6 types of surgical procedures. Some studies have shown that female residents who perform fewer cases with meaningful autonomy^22,23^ are less satisfied with surgical training^24^ and are more likely to leave training compared with male residents.^25,26,27^ Furthermore, female residents are significantly underrepresented among award recipients in general surgery residency programs.^28^ In a study on gender disparity in robotic surgical experience in a colorectal surgery training program,^29^ female residents had lower rates of console participation and fewer opportunities to complete total mesorectal excision than male residents. Furthermore, female attending surgeons offered equal opportunities for surgical experience to male and female residents, but male attending surgeons offered fewer opportunities to female residents.^29^ The majority of attending surgeons in Japan are male, and a prevailing gender bias among them may influence the surgical experience of residents.

A decline in the surgical experience of female surgeons may also occur because of pregnancy, childbirth, and childcare. Pregnancy during training not only leads to a reduction in the effort and period of training for female surgeons but also increases prejudice among male attending surgeons.^30^ Gastrointestinal surgery is one of the subspecialties with long working hours and many emergent surgeries. Therefore, it is possible that female residents informed their supervisors in advance that they would not choose gastrointestinal surgery, which may have affected the number of surgeries performed in this field.

After completing their senior residency training, surgeons are affiliated with the surgical department of their choice. This study’s finding that the number of operative procedures performed by both male and female surgeons decreased after senior residency may be attributed to the fact that a certain number of them went on to graduate school. Nevertheless, the fact that male surgeons had more experience than female surgeons for all 6 operative procedures, and that the more difficult the procedure, the greater the gender disparity, is important and cannot be ignored. However, it is noted that female surgeons performed surgeries for high-risk patients, as male surgeons did, in all the year groups, and there was no disparity in the postoperative mortality rate between the genders.

Pregnancy, childbirth, and childcare certainly have an impact on the surgical experience of female surgeons. The burden of childcare could be expected to decrease after several years, and the gender gap in surgical experience could become smaller; however, as observed in this study, this gap became larger with an increase in the number of years after registration. In particular, the disparity between male and female surgeons increased over the years for medium- and high-difficulty operations. In this case, the huge difference in the surgical experience of female and male surgeons cannot be explained by maternity leave alone. In Japan, after choosing a subspecialty, a surgeon’s place of work is often determined by the university medical office that they belong to.^31^ Professors of each department appoint medical staff, and currently, all but 1 of the professors in departments of gastrointestinal surgery across Japan are male. Thus, it is worth exploring whether a gender bias exists in the appointment of medical staff.

Based on the results of this study, our specific recommendations are as follows:

1. It is necessary for all surgeons to realize that there is a difference in surgical experience between male and female surgeons that cannot be explained by pregnancy and childbirth alone, and to discuss ways to improve this. The 2015 initiative of the Royal Australasian College of Surgeons may help consider future measures. The College has apologized for discrimination against female surgeons and has since established a pioneering educational program to bring about necessary cultural change.^32,33,34^
2. It is important to motivate the administrators of medical colleges and the heads of surgical departments in all hospitals to eliminate discrimination in surgical training and place surgeons in training facilities without gender bias. In the future, hopefully, data from the NCD can be utilized to ensure the proper allocation and training of surgeons.
3. With regard to female surgeons with children, Brown et al^35^ stated that “it is possible to accommodate childcare during training if there is appropriate institutional support”; therefore, excessive restrictions should be avoided. Furthermore, it is essential to create a flexible and efficient program by holding discussions with female surgeons with children and paying attention to their needs.
4. It is necessary to actively promote female surgeons to positions of decision-making and authority in the JSGS. It is necessary to consider introducing a quota system to determine the number and ratio of leadership positions based on gender, and a goal and timetable system to set achievement targets and timeframes for specific numbers.

In recent years, the proportion of women in gastrointestinal surgery has been increasing, with approximately 20% of the field currently comprising women younger than 30 years. The Gender Equality Working Group was established within JSGS in September 2020. In 2021, the JSGS set the goal of appointing a fixed percentage of women as chairpersons and program committee members for its 77th General Meeting. In the future, a similar move is expected regarding council members. Thus, the JSGS is carrying out several reforms under the current president’s leadership, and it is hoped that further action will be taken with a view to the future of digestive surgery.

### Limitations

This study has some limitations that should be noted. First, in calculating the number of operations per surgeon, the denominator was the number of JSGS members, which may not reflect the full population of gastroenterological surgeons in Japan, as some JSGS members were not participating in surgery during the study period. Second, information about the members’ marriage, pregnancy, and childbirth was not registered with the NCD or JSGS. Third, some residents were members of the JSGS but not necessarily aspiring gastrointestinal surgeons, which may have affected their surgical experience.

## Conclusions

This cross-sectional study found that female surgeons in Japan have less surgical experience than male surgeons. Furthermore, gender disparity in surgical experience tended to widen with years of experience for medium- and high-difficulty operations. These findings suggest that the overwhelming lack of women in surgical leadership and management positions was associated with the lack of equal opportunities for surgical training. It is necessary to build a system to eliminate gender disparity in surgical training and discrimination against female surgeons.
